# 3D Printing and Electrospinning of Composite Hydrogels for Cartilage and Bone Tissue Engineering

**DOI:** 10.3390/polym10030285

**Published:** 2018-03-08

**Authors:** Arianna De Mori, Marta Peña Fernández, Gordon Blunn, Gianluca Tozzi, Marta Roldo

**Affiliations:** 1 School of Pharmacy and Biomedical Sciences, University of Portsmouth, Portsmouth PO1 2DT, UK; arianna.demori@port.ac.uk (A.D.M.); gordon.blunn@port.ac.uk (G.B.); 2 Zeiss Global Centre, School of Engineering, University of Portsmouth, Portsmouth PO1 3DJ, UK; marta.pena-fernandez@port.ac.uk (M.P.F.); gianluca.tozzi@port.ac.uk (G.T.)

**Keywords:** composite hydrogels, electrospinning, 3D printing, bone, cartilage

## Abstract

Injuries of bone and cartilage constitute important health issues costing the National Health Service billions of pounds annually, in the UK only. Moreover, these damages can become cause of disability and loss of function for the patients with associated social costs and diminished quality of life. The biomechanical properties of these two tissues are massively different from each other and they are not uniform within the same tissue due to the specific anatomic location and function. In this perspective, tissue engineering (TE) has emerged as a promising approach to address the complexities associated with bone and cartilage regeneration. Tissue engineering aims at developing temporary three-dimensional multicomponent constructs to promote the natural healing process. Biomaterials, such as hydrogels, are currently extensively studied for their ability to reproduce both the ideal 3D extracellular environment for tissue growth and to have adequate mechanical properties for load bearing. This review will focus on the use of two manufacturing techniques, namely electrospinning and 3D printing, that present promise in the fabrication of complex composite gels for cartilage and bone tissue engineering applications.

## 1. Tissue Engineering

Defects that affect tissues such as cartilage or bone can be irreversible and become a clinical challenge. This is particularly true when these lesions are associated with conditions such as osteoarthritis and osteoporosis. Currently, it is estimated that 75 million people suffer of osteoporosis in Europe, USA and Japan [1], while 27 million are affected by osteoarthritis in USA only [2]. These numbers are certainly due to increase, as the population is aging, leading to higher healthcare costs. Current treatments for bone (i.e., autografts and allografts) present drawbacks such as limited availability of donor tissue, risk of infection and unsatisfactory lesion repair [3], whilst the use of bone graft substitutes are not as reliable as gold standard autograft. For small cartilage defects autologous chondrocyte implantation, mosaic pasty and autologous matrix-induced chondrogenesis can be used with variable success but for larger cartilage defects, often joint replacement is the only solution.

Therefore, new strategies are needed to repair damaged cartilage and bone tissue [4]. Tissue engineering is a promising interdisciplinary approach in this field: it aims at developing temporary 3D multicomponent scaffolds that mimic the natural tissue, working as a porous framework for the migration, adhesion and growth of cells to replace the damaged biological material. Ideally, a scaffold for tissue regeneration should have a highly interconnected porous network for the diffusion of nutrients and gases, have good mechanical properties in loadbearing conditions and degrade without producing toxic products with increasing formation of new tissue [3].

## 2. Challenges in Tissue Engineering of Soft and Hard Tissues

Tissue engineering has become one of the most commonly used approaches for cartilage and bone tissue repair [5,6,7,8]. Even though these two tissues are important constituents of the skeletal system, their structure and mechanics differ considerably. Bone is a hard and rigid tissue, whereas cartilage is soft, viscoelastic and flexible and these two tissues are different in several aspects [9]. In addition, according to the anatomic location and function, the same type of tissue can be heterogeneous displaying different anisotropic properties, biochemistry and cellular activity [10]. These characteristics complicate the fabrication of scaffolds that successfully mimic the structural and mechanical characteristics of the target tissue.

### 2.1. Cartilage

Cartilage is a strong and elastic connective tissue that covers the articulating surface of the bone in diarthrodial joints (articular cartilage) and is a structural component of the rib cage, ear, nose and other body components [11]. Three types of cartilage exist, according to the extracellular matrix (ECM) composition: elastic cartilage (if elastic fibres are present in the ECM), fibrous cartilage (if the matrix is rich in collagenous fibres) and hyaline cartilage if the matrix is predominantly composed of glycosaminoglycans, (GAGs). The latter, also known as articular cartilage, if found at the interface between the gliding bony surfaces in the articular synovial joints, provides a deformable low-friction surface that facilitates the movement of articulating bones and supports high dynamic compressive loads [12]. Mechanically, human cartilage presents the following characteristics: compressive modulus of 0.7–0.8 MPa, shear modulus of 0.7 MPa and tensile modulus of 0.3–10 MPa [13]. At the microscopic scale, human cartilage consists of an ECM, which can be mineralized and is produced and maintained by chondrocytes embedded within it. The hydrated ECM is composed of proteoglycans consisting of a core protein with covalently attached GAGs, mainly chondroitin sulphates, and collagen type II [14]. The GAGs are responsible for the cartilages’ ability to support high compressive loads, whereas the collagen II fibrils contribute to its high tensile strength and ability to tolerate shear stresses. Cartilage composition includes 80% of water, drawn into the collagen II fibrils by the hydrophilic proteoglycan complexes; this plays an important role in defining the tissue load-bearing function [12]. As cartilage is compressed, the extracellular matrix is compacted causing the efflux of water. As the cartilage is compacted more, the flow of water is reduced due to increasing drag increasing the hydrostatic pressure, which withstands the load. It is important to note that cartilage is avascular, which means that nutrients and cells infiltration is poor and wound healing, after injury or trauma, is hindered. In particular, fibrocartilage is formed to replace the native cartilage and this new tissue is functionally and biomechanically inferior to the native one. Current therapies to facilitate cartilage regeneration include autografting, microfractures and autologous chondrocyte implantation (ACI); however, they all present drawbacks and are unable to fully restore the functional hyaline cartilage, making long-term prognosis uncertain [15]. For instance, autografting causes donor site morbidity and is limited by donor tissue availability. Microfracture treatments can extend the damage to the surrounding tissue and stem cells implantation can still induce the formation of fibrocartilage. In this scenario, cartilage tissue engineered (CTE) scaffolds could be a turning point.

### 2.2. Bone

Bone is the main constituent of the musculoskeletal system [16] and differs from other connective tissues (i.e., cartilage, ligaments and tendons) in rigidity and hardness [17] due to high mineralization of its ECM [18]. Bone provides stiffness to the skeleton allowing for the shape of the body to be maintained, plays a role in the transmission of muscular forces for movement, affords protection to soft tissues within the cranial, thoracic and pelvic cavities as well as the bone marrow [19,20]. At the microscopic scale, bone is arranged in two architectural forms: trabecular and cortical bone. Trabecular bone represents 20% of the skeletal mass and forms the inner part of the bone, it presents high porosity (50–90%) and contributes to mechanical support in bones such as the vertebrae. Cortical bone, the dense outer layer of bone, comprises 80% of the weight of the human skeleton, and its function is to provide mechanical strength and protection [16]. Mechanically, the bone presents the following characteristics: the compressive strength for cortical bone ranges from 170 to 193 MPa and its elastic modulus is found to be in the range of 7–20 GPa. Trabecular bone, however, has a compressive strength ranging from 2 to 12 MPa and its modulus is in the range of 0.1–5 GPa [21,22]. Bone tissue presents a highly complex and hierarchical structure [23] which can be defined as a nanocomposite consisting of inorganic nanocrystalline hydroxyapatite (HAp), organic components (mainly type-I collagen) and water [24]. The nanocomposite structure is essential to provide the required compressive strength and high toughness of the bone [25]. Collagen fibres reinforced by HAp crystals form a tough and flexible nanostructured extracellular matrix, which supports adhesion, proliferation, and differentiation of bone cells (osteoblasts, bone lining cells, osteocytes, and osteoclasts) [19,26,27]. Bone constantly undergoes remodelling during life to help it adapt to loading conditions; remove old, microdamaged bone replacing it with new, mechanically stronger bone; and help to preserve bone strength [28]. This remarkably dynamic structure of bone displays exceptional regenerative properties [29]; however, non-union fractures, tumour resections and some musculoskeletal diseases can lead to critical size bone defects [30] that cannot heal spontaneously and require additional treatment before they can regenerate [31]. Bone is the most commonly transplanted tissue after blood [32]. Several therapeutic approaches including bone grafting procedures [33], implantation of different biomaterials [34] and application of hormones or growth factors have been investigated [35]. However, there is still no effective treatment for most cases [36,37]. The limitations of autografting and allografting were previously addressed [38,39]. Metal implants provide immediate mechanical support, but present limitations due to poor integration with the tissue, infection and fatigue fracture [40,41]. Furthermore, ceramic bone graft substitute materials present very low tensile strength and are brittle, restricting their use in locations of significant torsion, bending or shear stress [42]. In this perspective, bone tissue engineering (BTE) has emerged as a promising approach for bone reconstitution, overcoming the limitations of traditional implants [43,44,45].

### 2.3. Osteochondral Tissue

Osteochondral (OC) tissue is located at the interface between the osseous and the chondral tissue and it promotes their interplay; its role and location require a complex composition that includes cartilage, calcified cartilage and subchondral bone. The mechanical, structural and biochemical characteristics of the osteochondral tissue vary throughout. For instance, from a biochemical point of view, mineral content increases from cartilage to bone, while collagen and water concentration diminishes. Structurally, pore size, porosity and vascularization increase from cartilage to bone face. Mechanically, compressive modulus increases from cartilage to osseous tissue [46]. Osteochondral defects (OCDs) seem to play an important role in the genesis of joint diseases, such as osteoarthritis or osteochondritis dissecans. Moreover, subchondral bone includes unmyelinated free nerve endings which may cause pain in case of OC degeneration, due to the applied forces from surrounding tissues [47]. This said, it is fundamental to repair osteochondral defects to prevent joint destruction. However, OCDs are extremely difficult to treat due to the widely different features between articular cartilage, calcified cartilage and subchondral bone [48]. Osteochondral scaffolds should be designed to concurrently rehabilitate these three tissues all together. Several approaches have been studied to promote OC regeneration, such as debridement, bone marrow stimulation techniques, and the use of osteochondral allografts. However, these present strategies are affected by many drawbacks. For instance, microfractures may lead to degeneration of the repaired tissue and formation of the non-functional fibrocartilage. In the case of grafting techniques, there is lack of donor tissue or immunoreactions. Considering the complexity of this tissue, more research into osteochondral engineering is required [46,49].

## 3. Hydrogels as Tissue Engineering Scaffolds

Hydrogels have been widely investigated not only for tissue engineering applications, but also for drug delivery and wound dressing [4,50,51]. They are insoluble hydrophilic polymeric networks that can swell without disintegrating and absorb a high degree of water, up to several times their dry weight [52]. Their fully hydrated 3D structure resembles the extracellular matrix of native tissues, both physico-chemically and biologically. Moreover, their porous structure enables the transfer of nutrients and metabolites that are fundamental for cell growth. Hydrogels can be formulated from different natural and synthetic polymers such as alginate, chitosan, fibrin, hyaluronic acid (HA), poly(ethylene glycol) (PEG) and poly(ethylene oxide) (PEO). However, hydrogels often show inadequate mechanical performance, due to the interstitial liquid and its plasticizing effect, which make them too weak for applications in the musculoskeletal system [53]. For instance, most hydrogels have an elastic modulus ranging from kPa to MPa, whereas native bone has a modulus of ~1–20 GPa. Matching these properties is fundamental for two main reasons: (1) scaffolds must support loads and movements; and (2) cells respond differently to different stresses, such as compression, tension and shear [54]. Luckily, hydrogels are tuneable materials; their chemical modification and differential crosslinking allow achieving the desired properties for the proposed application. For example, it is possible to increase the hydrogel elastic modulus increasing crosslinks density inside the gel or combining two or more independent networks, known as interpenetrating networks. Numerous researchers have worked on finding ways to formulate hydrogel constructs with optimized mechanical properties [55,56,57], and several techniques have been employed in order to fabricate them [58]. Microfabrication techniques such as electrospinning and 3D printing have emerged as promising strategies for manufacturing complex hydrogel structures for tissue engineering applications.

## 4. Electrospinning

**Figure 1:**
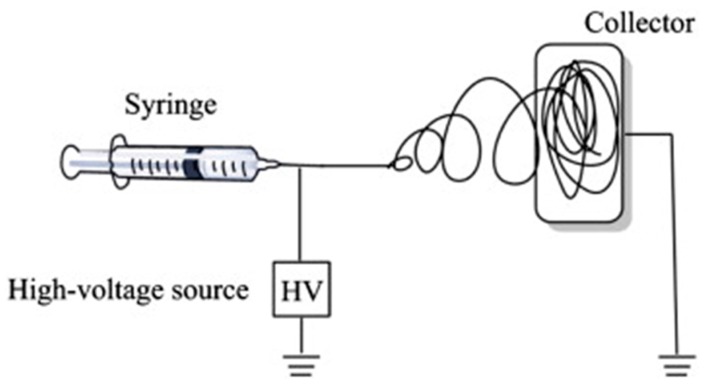
Schematic illustration of the basic setup for electrospinning. Reproduced from [62] with permission. Copyright (2015) Elsevier.

Electrospinning is a versatile, efficient, cheap and reproducible technique that can be used to produce 1D fibrous materials or composites with a wide range of diameters (from nm to mm), by applying an electrostatic force to a solution. Applications in tissue/organ repair and regeneration [59], drug delivery [59], medical diagnostic, protective fabrics against environmental [60] and infectious agents, and dental materials have all been studied [61]. A general electrospinning apparatus (Figure 1) consists of three parts: a high voltage power supply device; a syringe/capillary tube with a metallic needle; and a grounded metallic collector.

**Figure 2:**
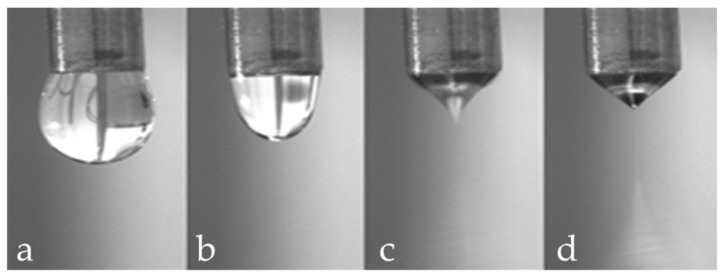
Effect of increasing applied voltage on the shape of the solution drop ejected by the needle: (**a**) normal dripping; (**b**) micro dripping; (**c**) intermittent Taylor cone; and (**d**) Taylor cone jet.

In a typical electrospinning process, a polymeric solution (or melt) is loaded into a syringe and ejected at a controlled rate, forming a drop (Figure 2a). Simultaneously, a high voltage (up to 2–30 kV, depending on the solution used) is applied, and a charged jet of the polymeric solution or melt is formed (Figure 2b–c). When the electrostatic repulsion starts to overcome the surface tension of the fluid, the hemispherical surface of the liquid will deform into a conical shape, called Taylor cone, at the tip of the needle (Figure 2d). Finally, the solution jet starts to evaporate, and the polymer solidifies creating a thin fibre jet that deposits on the grounded collector.

The electrospinning of fibres is relatively complex, as the product characteristics can be influenced by several parameters classed into three categories:

1. Solution characteristics (viscosity, surface tension and conductivity): the electrospinning technique relies on the uniaxial stretching of a charged jet, which, in turn, is significantly affected by changing the concentration of the polymeric solution. Generally, by reducing the polymer concentration, the fibre diameter is decreased. However, when the concentration of the polymeric solution is lowered to a critical value, known as entanglement concentration (Ce), beaded fibres are produced. If the concentration is too high no fibres are produced due to the excessive viscosity [63]. Solution conductivity is fundamental to optimize both fibre diameter and stability of the Taylor cone. If the solution has a low conductivity the fluid surface cannot be charged, and no Taylor cone can be formed. By increasing the charge on the surface of the droplet a Taylor cone is formed and the fibre diameter is decreased [64]. The conductivity of a polymeric solution can be controlled by the addition of salts. Moreover, the solvent has a crucial role in determining the characteristics of the solution; an ideal solvent must to be able to solubilize the polymer at the required concentrations and be sufficiently volatile to evaporate in the space between the needle and the collector. However, if the boiling point is too low, the solvent will evaporate too quickly causing the drying of the jet at the needle tip [65].
2. Process parameters (applied voltage, flow rate and tip to collector distance): these parameters influence the diameter and morphology of the fibres. As the feed rate increases, the charge density will decrease. Thus, by increasing the flow rate the diameter of fibres is increased and beaded morphology can be observed [64]. Fibres are formed just when the applied voltage is higher than the threshold voltage (the value depends on the solution). Generally, by increasing the voltage there is an increase of the electrostatic force on the solution and thus, a reduction of the fibre diameter. A critical distance between tip and collector is needed for the solvent evaporation and for the preparation of smooth and uniform electrospun fibres. Generally, the longer the distance, the thinner the fibres will be.
3. Environment conditions (humidity and temperature) [66,67]. Ideal environmental conditions must be found for better and improved fibre production. Ambient humidity and temperature can affect both the morphology and diameter of the fibres. Depending on the chemistry of the polymer [68], the fibre diameter can increase or decrease and no definitive comparisons with experimental data can be currently made.

**Figure 3:**
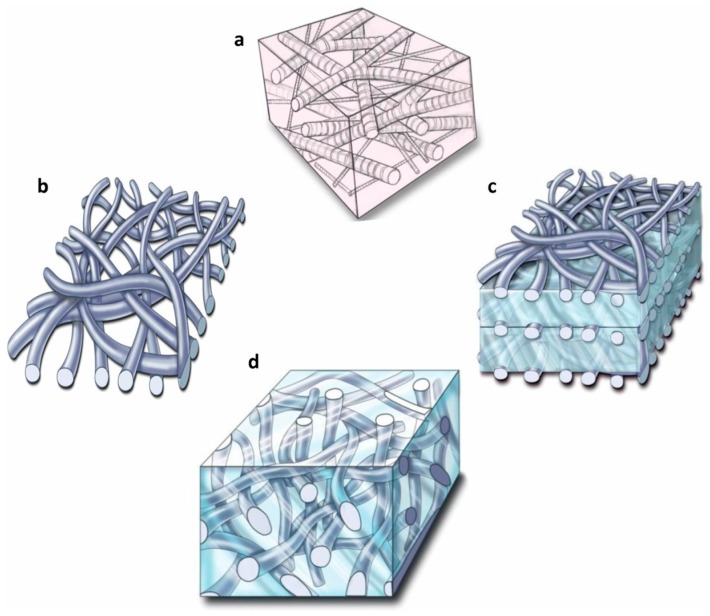
(**a**) Architectural framework of a native extracellular matrix (ECM); (**b**) electrospun 2D mat; (**c**) laminated hydrogel; and (**d**) fibres encapsulated into a hydrogel. Reproduced from [71] with permission. Copyright (2011) PMC.

To date, many electrospun scaffolds made of polymers and inorganic nanoparticles have been produced for tissue engineering applications. However, these electrospun scaffolds are in the form of 2D mats (Figure 3b) with tightly packed fibres that negatively impact cell infiltration and growth throughout the scaffold. Alternatively, scaffolds obtained by the amalgamation of fibres and hydrogels can be used to achieve better properties, such as a well-interconnected porous structures [69]. Different composite structures have been employed to fabricate fibrous hydrogels: laminated composites (Figure 3c), encapsulating fibres into the hydrogels (Figure 3d), injectable hydrogels, composite coatings and dual electrospun/electrospray composites [70].

### 4.1. Fibrous Hydrogels for Cartilage Tissue Engineering via Electrospinning

Articular cartilage plays an important role in load-bearing joints during dynamic loading. When damaged, it cannot heal naturally, and clinical treatments are necessary. Tissue engineering aims at temporarily replacing damaged articular cartilage with 3D scaffolds. Hydrogels are promising materials for cartilage regeneration when strategies to reinforce their structure are applied, for example combining them with electrospun fibres. Indeed, these scaffolds can emulate the natural ECM (for its porosity and water content) and possess improved mechanical properties due to the fibres ability to reorient under deformation, stiffening, strengthening and toughening the system. Thus, researchers have tried to optimize the mechanical properties of the hydrogels, studying different parameters such as the polymers used, fibre diameter and alignment, porosity and mono or multi layered scaffolds. 

Many synthetic and natural polymers have been used for electrospun fibres for cartilage regeneration, such as polycaprolactone (PCL), poly(lactic-*co*-glycolic acid) (PLGA), poly(l-lactic acid) (PLLA), poly(vinyl alcohol) (PVA), *Bombyx mori* silk fibroin and chitosan. Chitosan, an amino saccharide that is biodegradable and cytocompatible, with antibacterial and wound healing activities as well as tissue-adhesive features has been one the most studied polymers. This polymer can be easily obtained by alkaline treatment of chitin, a naturally occurring polysaccharide obtained from the exoskeleton of crustaceans. To overcome chitosan poor mechanical properties, Mirahmadi et al. [72] developed a chitosan hydrogel enriched with electrospun silk fibroin fibres. They fabricated two gels, one with homogeneously dispersed chopped degummed silk fibres (SC/GP-D) and one as a three-layered composite with a layer of electrospun fibres sandwiched between two layers of chitosan gel (SC/GP-L). Results showed that the mechanical properties were generally enhanced by silk and that the laminated gel presented both better compressive and Young’s moduli compared to chitosan only (3.1 times stiffer) even though the mechanical performances were not as good as the natural cartilage. On the other hand, the SC/GP-D scaffold was the best scaffold for cartilage formation (as shown by proteoglycan and collagen II content) among the studied hydrogels [72]. For future studies, a combination of both degummed fibres and nanofiber sheets could be examined to obtain improved mechanical properties. A second possibility to improve the mechanical characteristics of the gel is to incorporate individual and short electrospun nanofibers in hydrogel scaffolds, positioning them in a random way to favour an irregular orientation of the chondrocytes. Mohabatbour and his group [73] fabricated PLA fibres fragmented through aminolysis reaction to improve their hydrophobicity and cell-interaction abilities. They fabricated an alginate grafted hyaluronic acid (Alg-HA) incorporating fragmented PLA nanofibers. The nanofiber incorporated hydrogels had higher compressive modulus and lower swelling ratio than Alg-HA hydrogel alone. In this case, the composite was cytocompatible and the chondrocytes were able to maintain their functional properties producing GAGs and other extracellular molecules. This research highlighted that, to control gel fracture and strength, fibres can be differently oriented into a hydrogel.

Other studies have focused on introducing biological signals such as chondroitin sulphate (CS), hyaluronic acid, and collagen, into tissue-engineered scaffolds to encourage tissue specificity. Coburn et al. [74], for instance, fabricated poly(vinyl alcohol)-methacrylate (PVA-MA) fibrous scaffolds with or without chondroitin sulphate, a signal that has been shown to enhance chondrogenesis of mesenchymal stem cells. These hydrogels allowed for immediate cell infiltration, cartilaginous tissue formation and chondrogenic differentiation (as indicated by a higher cartilage specific gene expression). Finally, the addition of CS increased type II collagen deposition compared to PVA fibres alone [74]. 

More recently, solution electrospinning has been further developed into Melt Electrospinning Writing (MEW), this exploits a layer-by-layer process similar to other 3D printing technologies affording highly organised fibrous 3D structures in the micron scale. This process eliminates the need for organic solvents that can induce cell toxicity and avoids mechanical and electrical coiling of the fibres simplifying the manufacturing process. Using this technique, Bas et al. [75] produced a negatively charged proteoglycan matrix with a star-shaped poly(ethylenglycol)/heparin hydrogel combined with wet melted PCL fibres and deposited a 0°–90° crosshatch architecture with different network spacing. The best electrospun matrix had 600 μm spacing; its negative charge density and strong water retention capacity, provided by PEG crosslinked heparin, accurately mimicked the natural cartilage tissue in terms of electrochemical, mechanical and viscoelastic properties. The constructs presented high chondrocyte viability and allowed for cell differentiation under physiologically relevant loading.

Even though the most common strategy is to form fibrous scaffolds with embedded fibres within hydrogels, multilayer scaffolds constructed with fibres in different orientations have been investigated. For instance, Tonsomboon et al. [76] studied how different designs of laminated and non-laminated electrospun gelatin nanofibers in an alginate hydrogel could mimic the mechanical characteristics of the collagenous ECM. In particular they have fabricated single layer composites (a) with or (b) without a random orientation or multilayer composites with (c) unidirectional (where fibres had the same orientation), (d) cross-ply (where alternating layers were perpendicular) or (e) angle-ply orientation (where there were four different fibres orientations). Firstly, this work showed that nanocomposite hydrogels were stronger and tougher than single polymer hydrogels. Secondly, aligned fibres increased tensile strength without improving toughness. Thirdly, multilayer arranged nanofibers increased the toughness by two orders of magnitude when compared to the controls [76]. Therefore, this paper demonstrated that, by tuning different architectures of fibre reinforced and laminated composite hydrogels, it is possible to resemble the mechanical properties of the native tissue. Literature on hydrogels reinforced with electrospun fibres for cartilage regeneration is summarised in Table 1.

### 4.2. Fibrous Hydrogels for Bone Tissue Engineering via Electrospinning

**Figure 4:**
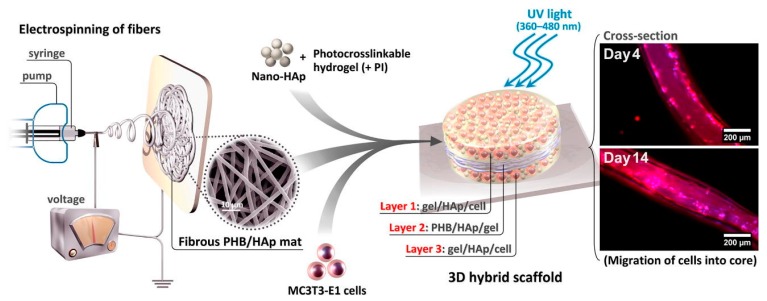
Schematic overview of the method to fabricate 3D cell-laden laminated hydrogels. Reproduced from [69] with permission. Copyright (2016) Elsevier.

Hydrogels have been suggested as possible scaffolds for bone regeneration, but their poor mechanical properties and low bioactivity make them inappropriate for hard tissue. One strategy is to reinforce the gel with electrospun fibres, but only a few studies have been reported on fibrous scaffolds for application in bone tissue engineering. Mehdi-Sahad et al. fabricated a 3D cell-laden three-layered hybrid scaffold (Figure 4), incorporating a 2D mat layer of poly(hydroxybutyrate) (PHB) and nano-hydroxyapatite fibres (diameter 2.0 ± 0.2 μm) between two layers of methacrylated gelatin/HAp. As expected, the introduction of PHB/HAp fibres enhanced the mechanical strength (tensile modulus 7.0 ± 1.2 MPa, tensile strength 329 ± 18 kPa for the hybrid scaffold) and matrix mineralisation while the hydrogel provided a biocompatible scaffold for cell penetration and proliferation. Even though the mechanical properties were improved in comparison to the hydrogel only, they were still inferior to the natural tissue. Thus, Sadat-Shojai and his group [69] suggested increasing the thickness of the electrospun mat located at the centre of the scaffold, however this approach would lead to a denser electrospun mat, with decreased porosity that would potentially hindered cell penetration into the electrospun centre.

**Figure 5:**
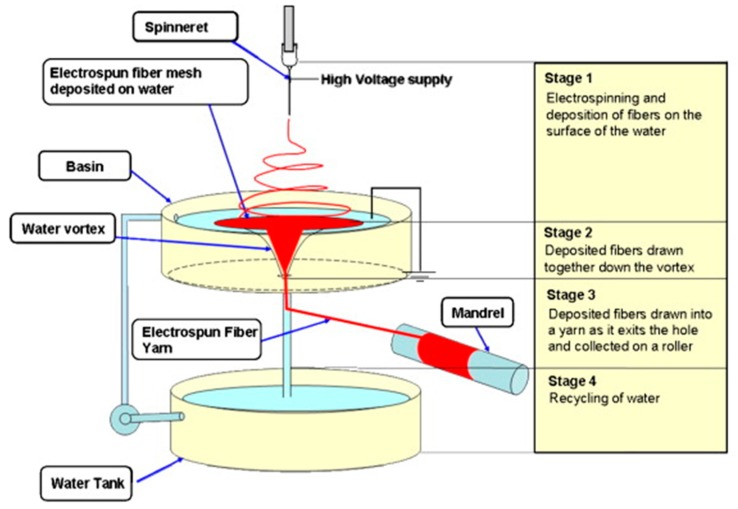
Schematic of the steps for the formation of nanoyarns using a water vortex. Reproduced from [82] with permission. Copyright (2007) Elsevier.

A further problem encountered in bone grafting procedures is the development of infection, inflammation and pain, intrinsically linked to any invasive procedure. However, due to poor vascularity of the bone tissue, osteomyelitis is often difficult and costly to treat. For this reason. injectable systems for minimally invasive procedure (MIP) have been developed. Calcium phosphate cements are among the most used cements (e.g., Hydroset Accell 100™) for bone regeneration as they are bioactive, and they have the ability of self-hardening. However, they are brittle, difficult to inject and present limited porosity, therefore research has been focusing on replacing the cements with injectable hydrogels. Liu et al. [81] have produced a biomimetic bone substitute made of chopped poly(l-lactide-*co*-ε-caprolactone) nanoyarns manually incorporated in a collagen hydrogel before gelation. Interestingly, to obtain well aligned nanoyarns, a water vortex was used as collector, instead of more traditional systems as rotating drums or dual metal collection rings (Figure 5). As a result, they obtained massive continuous nanoyarns with homogenous diameters (16 ± 4 μm). Furthermore, the cut nanoyarns were short enough to avoid the formation of entanglements when they were mixed with collagen solution. Results showed again that the incorporation of nanoyarns improved the mechanical properties of the hydrogel, without interfering with the cell proliferative ability of collagen.

**Figure 6:**
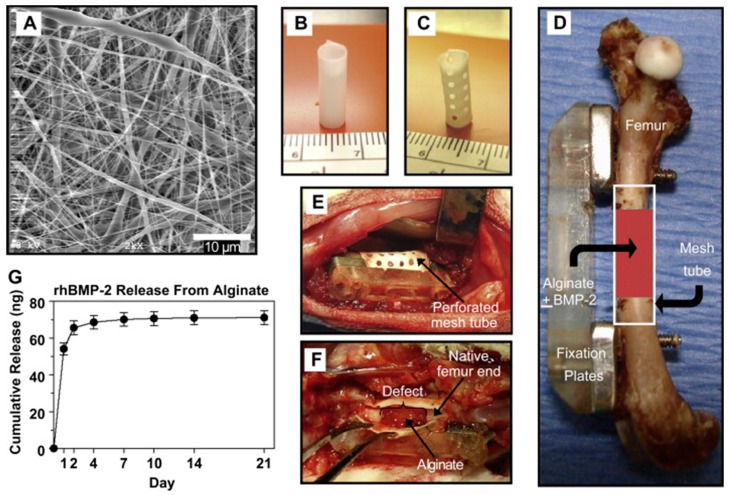
(**A**) SEM images of electrospun nanofiber mesh; (**B**) tubular implant without perforations; (**C**) tubular implant with perforations; (**D**) stabilized femur defect with implant; (**E**) bone defect, after placement of a perforated mesh tube; (**F**) alginate hydrogel was still present after 1 week, in vivo; and (**G**) release of rhBMP-2 from alginate hydrogel. Reproduced from [83] with permission. Copyright (2010) Elsevier.

Another approach for promoting osteogenesis in comparison to just polymeric hydrogels is to include growth factors inside the matrix. The scaffold needs to work as a delivery system promoting a sustained release and improved local retention. Kolambkar et al. [83] introduced electrospun nanofiber mesh tubes as a guide for rat bone regeneration in a segmental bone defect to deliver recombinant bone morphogenetic protein-2 (rhBMP-2) (Figure 6G). The PCL nanofibers (Figure 6A) had diameters ranging from 51 to 974 nm, with high porous meshes (80–90%). The thick nanofiber meshes were able to be wrapped tightly around a steel mandrel and glued to form a tube (of 5 and 13 mm length) that was finally put in a mouse bone defect (Figure 6D,E), as they were (Figure 6B) or after being perforated (1 mm diameter perforations) (Figure 6C). Then, 125 μL pre-gelled 2% alginate with or without 5 μg rhBMP-2 were injected in the tube lumen. As control groups, they examined the nanofiber mesh alone and in combination with alginate hydrogel. Results showed that the systems containing meshes + hydrogel + rhBMP-2 produced substantial bone formation and complete defect bridging, while the controls did not exhibit any significant bone repair response. Indeed, defects were bridged by 12 weeks with densely packed, cellular mineralized tissue for both perforated and not perforated meshes containing alginate loaded with rhBMP-2. However, micro-computed tomography (μCT) revealed that perforations in mesh tubes enhanced bone formation at earlier stages in comparison to the scaffolds without perforations. Moreover, samples implanted with both perforated mesh tube and rhBMP-2 containing alginate were the only one presenting mechanical performances, in extracted femora at 12 weeks, statistically similar to the ones of natural bone. They attributed this phenomenon to the fact that the perforations allow sufficient vascularization to develop, while limiting soft tissue ingrowth [83].

### 4.3. Fibrous Hydrogels for Osteochondral Engineering via Electrospinning

The challenge in the development of OC scaffolds is that they should be able to replicate the complexity of this tissue, and therefore restore cartilage, intermediate calcified cartilage and bone tissues, all together. To achieve this result, composites scaffolds should be able to recruit mesenchymal cells from the bone marrow. Moreover, they must present a stratified structure in order to mimic the three different functional layers of OC tissue [46]. Hydrogel/fibre 3D composites have a great potential to mimic this complexity; however, to date, literature on graded or non-graded hydrogels with electrospun fibres for osteochondral regeneration is scarce. Single-phase composites have been used for instance by Coburn et al.; they fabricated poly(vinyl alcohol)-methacrylate (PVA-MA) fibrous scaffolds with or without chondroitin sulphate, a signal that has been shown to enhance chondrogenesis of mesenchymal stem cells. These hydrogels, implanted into rat osteochondral bone defects, allowed for immediate cell infiltration, cartilaginous tissue formation and chondrogenic differentiation, as indicated by a higher cartilage specific gene expression. Furthermore, the addition of chondroitin sulphate increased type II collagen deposition compared to PVA fibres alone [74]. Filovà et al. prepared a PVA/liposomes blend that was electrospun and finally incorporated into a fibrin/type I collagen/fibrin composite hydrogel. Compressive tests showed the addition of nanofibers improved the mechanical properties of the composite gel as predicted. Moreover, once implanted into osteochondral defects of miniature pigs, the composite scaffold had better osteochondral regeneration towards hyaline cartilage and/or fibrocartilage compared with the controls that were mainly filled with fibrous tissue [84].

## 5. 3D Printing

Three-dimensional (3D) printing refers to manufacturing techniques in which 3D models are built in a computer-controlled layer-by-layer process [85]. It should be clarified that it is common to use the term “3D printing” in literature and mainstream media when referring to all Rapid Prototyping (RP) techniques; however, 3D printing also refers to a particular RP inkjet-based technology. The main advantage of 3D printing techniques in tissue engineering relies on the possibility of generating 3D scaffolds with a precise control over the internal architecture [86,87]. Furthermore, fabrication of scaffolds with a complex, subject-specific geometry by modelling data acquired using different imaging techniques such as magnetic resonance imaging (MRI) or computed tomography (CT) scans can be achieved [88]. Around 20 different 3D printing techniques have been applied to the biomedical field [86]; however, not all of them are compatible for the processing of hydrogels. For an extensive review in the hydrogel-rapid prototyping for tissue engineering the reader can refer to a recent publication by Billiet et al. [89]. 

Briefly, 3D printing of hydrogels can be divided in three main methods: laser-based, nozzle-based, and inkjet printer-based systems, depending on the stimuli employed to assist the printing process and deposition of the material. Despite the differences in material deposition mechanism employed in the different techniques, the typical apparatus includes a hydrogel reservoir from which the material is transferred in a controlled manner to an ejection system and a collection platform. Nozzle- and inkjet printers sequentially deposit material, while laser-based or laser-assisted systems are based on photopolymerization of the pre-deposited material irradiated by light energy in specific predefined patterns [90,91,92]. Nozzle-based or extrusion systems rely on a 3D dispensing process in which the hydrogel is extruded through a nozzle driven by compressed air or a piston/rotating screw [93,94,95]. Electrical signals are used to control the ejection of individual droplets and/or direction of a sequence of droplets [96,97]. 

**Figure 7:**
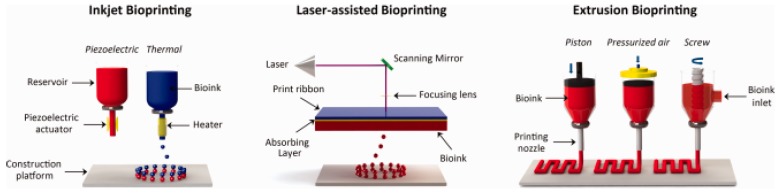
Illustration of 3D bioprinting technologies based on the mechanism used to assist the deposition of the bioinks and its main components; (**Left**) Inkjet bioprinters eject small droplets of cells and hydrogel sequentially to build up the scaffold; (**Middle**) Laser bioprinters use a laser to generate the local ejection of small droplets from a donor ribbon coated with the bioink; (**Right**) Extrusion bioprinters uses pneumatic of mechanical forces to continuously extrude the bioink through a nozzle. Reproduced from [100] with permission. Copyright (2015) Wiley-VCH.

These conventional 3D printing techniques are used to print cell-free 3D scaffolds for use in surgery [98]. In recent years, 3D bioprinting has gained popularity in tissue engineering to allow the direct one-step fabrication of 3D scaffolds containing biomaterials, cells and other biochemicals in the same structure. The working principles of 3D bioprinting are similar to conventional 3D printing techniques (Figure 7). The main difference lies on the deposition of the hydrogels together with small units of cells. For a thorough review on the basic principles of bioprinting, readers can refer to Mandrycky et al. [99].

**Figure 8:**
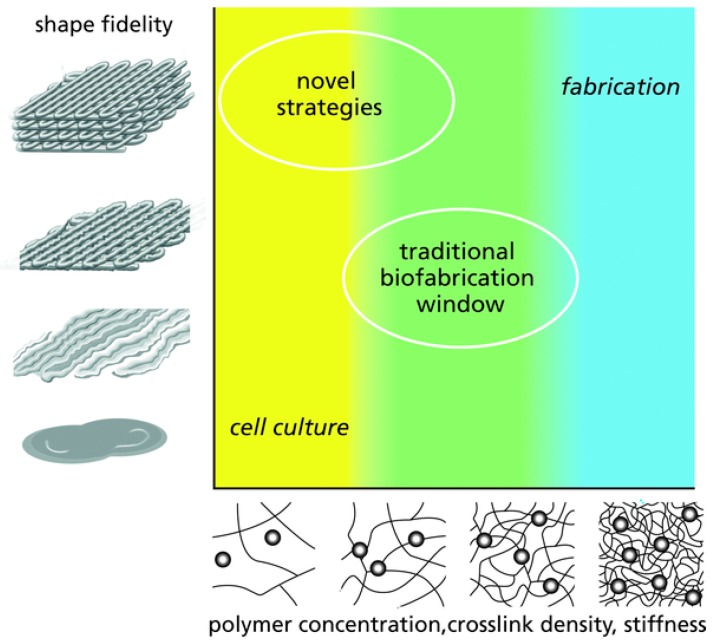
Schematic of the challenges for engineering bioinks suitable for 3D printing. Optimal shape fidelity can be typically achieved with stiff hydrogels (**top right**), however, this dense network limits cell viability. Contrarily, cell survive best in soft hydrogels, but shape fidelity cannot be achieved (**bottom left**). Therefore, a compromise between biological and fabrication properties must be done (**middle**). Novel strategies aimed at obtaining high shape fidelity with cytocompatible hydrogels. Reproduced from [105] with permission. Copyright (2009) Wiley-VCH.

Hydrogel inks used in 3D printing fabrication methods can be formulated from injectable, shear-thinning hydrogels [101], as they are required to flow under low pressures, gel quickly, and maintain their shape after build up [102]. When the hydrogel inks contain cells and/or biochemicals for the use in bioprinting, they are referred as bioinks [103]. The design of hydrogel inks for 3D printing starts with the formulation of a polymer solution that forms a connected network soon after printing. The printed network can be physically or chemically cross-linked as a response to external stimuli (e.g., temperature, light, and ion concentration) [104]. The development of hydrogel inks suitable for bioprinting (both fabrication and cell culture), remains a challenge [105]. Whereas stiff hydrogels containing high concentration of polymer are needed for optimal shape fidelity, these highly dense networks limit cell migration, growth and differentiation [106,107]. Conversely, cells grow better in soft hydrogels, which are too watery to maintain the desired shape (Figure 8). Maintaining shape conformity may compromise the biological competence and the clinical outcomes of the printed structures. Therefore, despite the advances in 3D printing technologies that allow researchers to design and fabricate complex structures, the lack of suitable bioinks for tissue engineering is restricting the progress in the field and its translation to clinical practice. Initially, 3D printing technologies focused on the use of pure polymers; however, as the technology advances, the development of novel composite-hydrogels for 3D printing are becoming increasingly popular, aiming at enhancing properties such as printability, mechanics and bioactivity [108,109,110,111].

### 5.1. 3D Printing of Hydrogels for Cartilage Tissue Engineering

Current scaffolds of 3D printing of hydrogels for CTE are mainly based on two different approaches: direct printing from hydrogels and hybrid printing from composite-hydrogels [112]. The advantage of using bioinks composed of a unique hydrogel is based on their simpler printability process compared to hybrid bioprinting and their physiological crosslinking conditions. Once again, when a high-level of printability is needed from the bioink, the mechanical properties of the 3D scaffolds are commonly weak [113], but the use of composite-hydrogels or the combination of a polymer network with bioinks can offer enough mechanical performance to support the 3D structure, although it may reduce the bioactivity.

**Figure 9:**
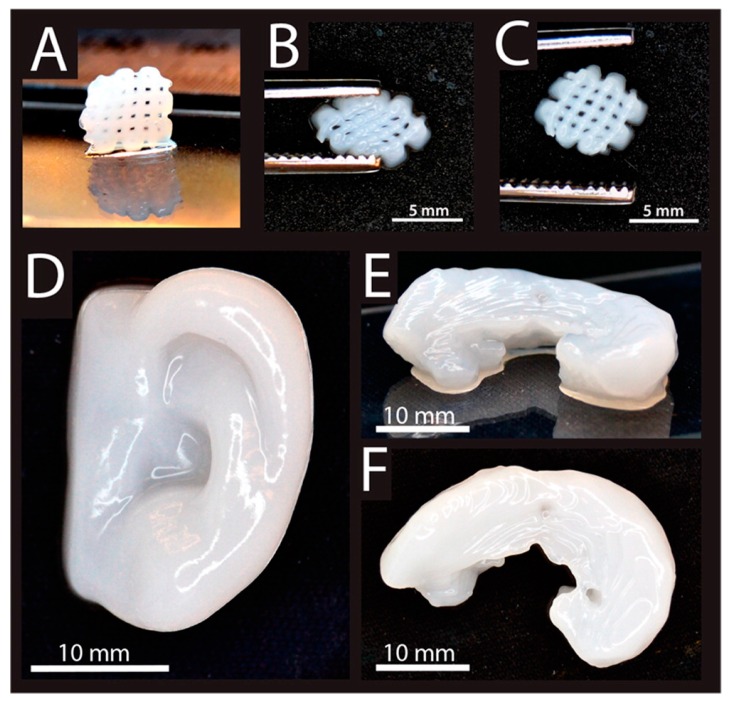
3D printed constructs made of a composite hydrogel (alginate + nanofibrillated cellulose) seeded with human chondrocytes. (**A**) 3D printed small grids (7.2 × 7.2 mm^2^). Deformed grid during (**B**), and after (**C**) squeezing. (**D**) 3D printed human ear. (**E**, **F**) 3D printed sheep meniscus. Reproduced from [115] with permission Copyright (2015) American Chemical Society.

Both natural and synthetic hydrogels have been used for CTE applications using 3D printing, where stem cells and chondrocytes are among the most common cell sources used in cartilage bioprinting [114]. Alginate has been extensively used as a bioink due to its rapid crosslinking. You et al. [113] successfully printed a porous cell-laden hydrogel scaffold using sodium alginate impregnated with ATDC5 chondrogenic cell lines or primary chick chondrocytes as a bioink. The resulting scaffolds supported cell survival (85% cell viability), proliferation and ECM deposition of chondrogenic cells in vitro, however the compressive modulus was considerably low (20–70 kPa) compared to human cartilage (700–800 kPa). The compressive modulus was enhanced (75–250 kPa) in Markstedt et al. [115] by combining cellulose nanofibers with alginate. As a result, the printed complex scaffolds supported the culture of human chondrocytes (73–86% viability after one and seven days) as shown in Figure 9. The same bioink formulation (nanofibrillated cellulose/alginate) in combination with human chondrocytes and MSCs was found to promote in vivo chondrogenesis after subcutaneous implantation of the printed constructs in mice [116,117], suggesting the potential of 3D bioprinting of human cartilage for clinical applications. On the other hand, cell viability (80–96%) and cartilage ECM deposition was improved by Kesti et al. [118] by developing a cartilage-specific bioink based on a blend of gellan and alginate and incorporating cartilage ECM particles and seeded with bovine articular chondrocytes. 3D scaffolds were successfully printed with good mechanical properties (tensile modulus: 116–230 kPa) and complex shapes (i.e., meniscus, intervertebral disks and nose).

Hyaluronic acid (HA) is gaining popularity as a bioink for CTE because of its viscoelastic and bioactive properties [119]. However, HA on its own has poor mechanical properties and it is therefore necessary to add other materials to improve printability and performance., Muller et al. [120] and Pescosolido et al. [121] demonstrated increased viability of chondrocyte cells by adding acrylated Pluronic and a dextran derivate to HA.

Gelatin has also shown excellent biocompatibility, but due to its low viscosity it is hard to print [122]. Therefore, gelatin is usually modified with an acrylate or methacrylate agent [123,124]. Gelatin-methacrylamide hydrogels (GelMA) have been extensively used to produce bioinks for CTE [125,126,127]. Schuurman et al. [127] demonstrated that introducing HA increased the printability and bioactivity of the 3D printed scaffolds, with high chondrocyte cells viability (82% after 3 days). Costantini et al. [126] showed that incorporating HA-methacrylate also enhanced the mechanical properties (compressive modulus ranging from 48 kPa for GelMA to 100 kPa for the composite bioink). On the other hand, Levato et al. [125] investigated different cell-sources to impregnate the GelMA-based hydrogels. They concluded that the use of articular cartilage-resident chondroprogenitor cells (ACPCs) as an alternative or in combination with chondrocytes and mesenchymal stromal cells (MSCs) supported the formation of 3D cartilage scaffolds in vitro.

Poly(ethylene-glycol) (PEG) is one of the most common synthetic hydrogels used for 3D printing in CTE, showing higher mechanical properties compared to natural hydrogels. Cui et al. [128] successfully 3D printed PEG-dimethacrylate (PEGDMA) with human chondrocytes reaching a compressive modulus of 396 kPa and high cell viability (89%). In addition, Gao et al. [129] combined PEG with GelMA and both hydrogels were printed together with human MSCs, demonstrating an improvement of the mechanical properties after chondrogenic differentiation.

The relative weak mechanical properties of all the above-mentioned hydrogel constructs considerably limit their application. To overcome this problem, alternating the printing of bioinks and thermoplastic polymers fibres is becoming more popular in CTE [130]. 3D printed scaffolds have been created by combining the deposition of a stiff polymer (polycaprolactone, PCL) and cell-laden hydrogel (alginate) [130,131,132], with chondrocyte cell viability varying from 70% to 85%. In vivo studies by Kundu et al. [131] showed enhanced cartilage ECM deposition by addition of transforming growth factor-β (TGFβ). Furthermore, Schuurman et al. [130] reported a compressive modulus of 6000 kPa of the printed hydrogels constructs.

### 5.2. 3D Printing of Hydrogels for Bone Tissue Engineering

Natural biopolymer hydrogels are excellent bioinks for 3D printing, due to their easily adjustable materials characteristics such as viscosity or gelation kinetics as well as their capacity to provide biocompatibility resulting in a consistency similar to the soft tissue matrix [133], however their weak mechanical properties limits the support of osteogenic differentiation and therefore their use for BTE [134,135]. Adjusting the scaffold composition is essential for the fabrication of bone tissue constructs. Composite hydrogel-based materials consisting of an hydrogel phase mimicking the organic part of the bone (mainly collagen type I) and a mineral phase representing the mineral content of bone (mainly hydroxyapatite) [136,137,138] can enhance the mechanical properties of the 3D scaffolds and their regenerative potential [139,140]. Natural (e.g., collagen, alginate, chitosan, HA, gelatin, and agarose) and synthetic (PEG) hydrogels have been used as bioinks for BTE applications, with the addition or not of inorganic particles (e.g., HAp).

HA is abundantly present in bone ECM, where it gives mechanical support. When it is modified with methacrylate groups, resulting in methacrylated HA (MeHA), printability and rigidity improve while maintaining good biocompatibility [141]. Poldevaart et al. [141] showed that human bone marrow derived MSCs survival (64% after 21 days) and osteogenic differentiation, measured by quantification of calcium deposition, were successfully achieved in 3D printed MeHA scaffolds; however, the elastic modulus of the hydrogel was very low (10.6 kPa) compared to bone tissue. MSC osteogenic differentiation and mechanical properties were enhanced [142] by combining agarose hydrogels with collagen type I. Different combinations of agarose-collagen were tested showing high cell viability (over 98%) and a compressive modulus ranging from 18 to 89 kPa. The less stiff hydrogel exhibited a higher MSC osteogenic differentiation in vitro. In vivo bone matrix formation was observed [143] after implantation of 3D printed alginate-gelatin scaffolds seeded with human adipose-derived stem cells (hASCs). Despite the good biocompatibility and osteogenic differentiation, using 3D printed hydrogel scaffolds without the addition of any inorganic particle notably affects the mechanical properties. 

**Figure 10:**
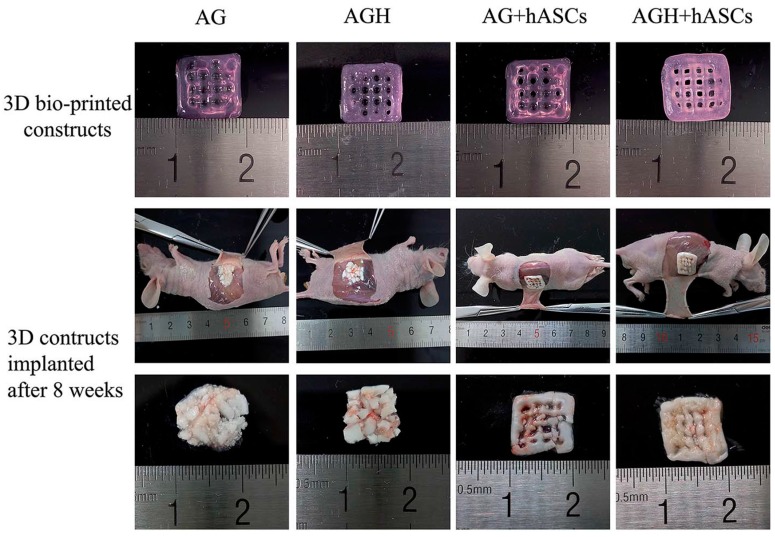
3D bio-printed constructs made of alginate/gelatin (AG) and alginate/gelatin/nano-HAp (AGH) mixed with human adipose-derived stem cells (hASCs) before and after implantation showing osteoinduction. Constructs were implanted into the back sub-cutaneous area of nude mice and harvested eight weeks after surgery. Larger bone formation was observed in the constructs containing HAp. Reproduced from [140] with permission, Copyright (2016) Royal Society of Chemistry.

Early attempts to enhance the performance of the printed hydrogels by incorporating HAp particles by Ang et al. [144] used a chitosan-HAp composite and showing cell biocompatibility. However, osteogenesis and mechanics were not evaluated, and cells were not printed together with the hydrogels. More recently, 3D printed cell-laden scaffolds using different hydrogel formulations (e.g., chitosan, alginate, and gelatin) with HAp particles have been studied [145,146]. The incorporation of HAp particles significantly improved the mechanical strength of the hydrogels and promoted osteogenic differentiation in vivo [140,145,146,147], making them suitable for repairing bone tissue defects (Figure 10).

Although hydrogels and composite-hydrogels have shown to be suitable for application in low load-bearing bone defects, adequate mechanical properties are still needed. In this perspective, hybrid scaffolds combining synthetic polymer scaffolds and cell-laden hydrogels are a promising area to explore. PCL is commonly used for bone scaffolds due to its high mechanical strength [98], but it presents limited cell affinity [148]. To improve cell proliferation and enhance the osteogenesis of PCL 3D printed scaffolds, Dong et al. [149] integrated them with bone marrow MSCs-laden chitosan hydrogel, achieving a compressive strength of the hybrid scaffolds of about 6.7 MPa (similar to trabecular bone). Osteogenesis was enhanced by incorporating chitosan into the PCL scaffolds and in vivo bone formation was found after implantation of the seeded scaffolds in a mice model [149]. Vascularization of 3D printed bone constructs was studied by Kang et al. [150] and Kuss et al. [151]. Cell laden hydrogels were hybrid bioprinted together with a PCL frame. Bone and vessel formation was observed in vitro and in vivo, resulting in promising results for the regeneration of bone defects. 

Works involving the use of 3D printing in the fabrication of scaffolds for bone and cartilage regeneration are summarised in Table 2 and Table 3. Even if these are not exhaustive lists of publications, they highlight the progress done on the front of providing viable cells for implantation. However, in vivo work is so far limited and needs to be explored more widely to bring these strategies closer to clinical translation.

### 5.3. 3D Printing of Hydrogels for Osteochondral Tissue Engineering

3D printing of hydrogels has shown great potential for the production of customized scaffolds in cartilage and bone tissue engineering, as previously described. Because of its ability to fabricate 3D constructs with complex shapes by depositing cell laden hydrogels at desired locations, 3D printing also results in a promising technique for the fabrication of gradient scaffolds with hydrogels stacked in a multilayer manner [152]. This unique capability enables to expand the use of 3D printing to the efficient regeneration of osteochondral tissue, providing a scaffold that favours integration between the chondral and the osseous phases for osteochondral defects repair. 3D printed constructs for osteochondral tissue regeneration are usually built in a bilayer fashion, by employing different bioink formulations for the subchondral bone and the cartilage zone. Early attempts were done by Fedorovick et al. [153] encapsulating human chondrocytes and osteogenic progenitors in alginate hydrogel, biofabricating 3D scaffolds with different parts for both cell types. Distinctive tissue formation at defined locations was observed both in vitro and in vivo, however, the scaffolds presented low mechanical strength (Young’s modulus < 7.6 kPa) and a limited height of the construct could be achieved. Anatomically relevant size bilayered scaffolds were fabricated by Levato et al. [154] combining two different bioinks: GelMA-Gellan Gum with PLA microcarriers (MCs) seeded with MSCs for the bone and without MCs for the cartilage layer. The MC laden region improved the compressive modulus (25–50 kPa) of the hydrogel constructs and supported osteogenic differentiation and bone matrix deposition by MSCs, suggesting the potential of the use of MCs-based biofabrication for osteochondral tissue engineering. An efficient osteochondral gradient scaffolds was fabricated using a novel laser-based 3D printer by Castro et al. [155] and human MSCs osteogenic and chondrogenic differentiation was enhanced through the incorporation of tissue specific nano-HAp with different concentrations of PEG-diacrylate (PEG-Da) for the porous osseous layer and the transitional calcified cartilage layer, and TGF-β1 added to the PEG-Da for the solid cartilage layer. It is known that hydrogels derived from natural ECM can enhance tissue regeneration by providing biochemical signals inducing cellular differentiation and migration [156,157]. In this sense, HA and collagen type I (Col-I), which are the major organic ECM components of cartilage and bone, respectively, were combined in a 3D printed scaffold fabricated by Park et al. [158]. A bilayer construct was 3D printed encapsulating chondrocytes in a HA hydrogel for the cartilage zone and osteoblast in a Col-I hydrogel for the bone area within a PCL printed framework. Viability and function of each cell type were well maintained up to 14 days in vitro. A validation of the potential of a 3D printed bilayer construct for osteochondral tissue regeneration using an in vivo animal model was reported by Shim et al. [159]. A subchondral bone layer was fabricated by dispensing a solution of atelocollagen with human turbinate-derived MSCs onto a PCL framework, whereas a solution of cucurbit [6] uril-HA and 1,6-diaminohexane-conjugated HA was dispensed into the PCL matrix for the superficial cartilage layer. 3D printed scaffolds were implanted in the knee joints of rabbits. Neo-cartilage was observed in the cartilage region and new bone formation in the subchondral bone region at eight weeks post implantation.

## 6. Future Trends

Electrospinning and 3D printing both have a great potential in the fabrication of complex structures such as those required for tissue engineering of bone, cartilage and osteochondral tissue. Combining the two could also overcome some of the limitations of the individual methods such as the tight intertwining of electrospun fibres that limits cell migration and the limited resolution of some 3D prototyping methods. The first reports of materials obtained by combining these two fabrication methods have only emerged in recent years [160]. More specific reports on their combined use in the manufacturing of tissue engineering scaffolds for bone, cartilage and osteochondral tissues are still limited. Yu et al. prepared PCL printed meshes that were infused with homogenised electrospun PCL/gelatine fibres crosslinked with glutarhaldehyde [161]. The work showed that combination of the mechanically competent 3D printed mesh with the biocompatible nanofibers can be exploited to obtain the ratio of porosity and the pore size that is most advantageous for cell migration and proliferation. Naghieh et al. fabricated scaffolds with alternating layers of 3D printed PLA mesh and gelatin/forsterite electrospun fibres [162]. These hierarchical structures presented appropriate mechanical behaviour for applications in bone tissue engineering with additional bioactive properties. These first reports demonstrate the potential of using a combinatorial approach, where 3D printing and electrospinning can afford additive advantages to the use of a single technique. The translational potential of this strategy must be now explored.

## 7. Conclusions

Hydrogels and their combination with other biomaterials are very attractive in tissue engineering applications targeting both soft and hard tissue replacement, with specific relevance to orthopaedics. To achieve complex hydrogel constructs with the necessary requirements to substitute the specific tissue and help regeneration, two manufacturing techniques are currently considered the most promising in the field: electrospinning and 3D printing. This review considers the most recent advances of hydrogel-based scaffold production using such techniques applied to cartilage and bone tissue engineering. As a result, a variety of materials can be used to fine-tune composite implants to achieve the desired mechanical and biological properties. However, as it stands, both electrospinning and 3D printing of hydrogel composites is still limited by the inability of selectively assign materials to reproduce tissue geometry and properties with required resolution and further work is needed in this sense, particularly to target tissue transition at the interface between bone and cartilage.

## Figures and Tables

**Table 1:** Publications on electrospun fibres reinforced hydrogels for cartilage regeneration.

Fibre(s)	Hydrogel	Fabrication method	Mechanical properties	Cytocompatibility	Reference
PCL or CSMA/PVAMA	PEG-diacrylate	Fibres mixed with the hydrogel	Not reported	Chondrogenic differentiation	[71]
Polyacrylonitrile	Alginate-polyacrylamide	Sandwich-like structure	Young modulus: 3.4 MPa	Not reported	[77]
PDLA/PLLA or PDLA/PCL	Chitosan	Fibre infiltrated with hydrogel	Compressive modulus: 2–12 MPa	Cartilage ECM deposition	[78]
PCL	GelMA and GelMA/HAMA	Fibres infiltrated with hydrogels	Compressive modulus: 20–1500 kPa	Not reported	[79]
PCL	PEG-heparin	Fibres infiltrated with hydrogel	Compressive modulus: 10–1500 kPa	Cell viability > 80% Chondrogenic differentiation	[75]
PLA	Alginate-graft-hyaluronate	Hydrogel mixed fibres and gelled	Compressive modulus: 3–5.4 kPa	Cell viability > 85% Chondrogenic differentiation	[73]
SIlk	Chitosan	Sandwich-like structure	Compressive modulus: 0.5–0.6 kPa	Cell viability > 90% Chondrogenic differentiation	[72]
PCL	Alginate or alginate sulphate	Hydrogel pipetted onto the scaffolds	Shear modulus: 0.5–5 kPa	Cartilage ECM deposition	[80]

**Table 2:** Overview of publications on 3D printing of hydrogels for cartilage tissue engineering.

Material(s)	Cell source(s)	Printing method	Mechanical properties	Cytocompatibility	Reference
Sodium alginate	ATDC5 chondrogenic cell line	Inkjet bioprinting	Compressive modulus: 20–70 kPa	~85% cell viability Cartilage ECM deposition	[113]
Alginate with cellulose nanofibers	Human nasoseptal chondrocytes	Inkjet bioprinting	Compressive modulus: 75–250 kPa	73–86% cell viability	[115]
Alginate with cellulose nanofibers	Human nasoseptal chondrocytes	Extrusion bioprinting	Compressive stress: 15–88 kPa	Cartilage ECM deposition	[116]
Alginate with cellulose nanofibers	Human nasoseptal chondrocytes and MSCs	Extrusion bioprinting	Not reported	Chondrogenic differentiation Chondrocytes proliferation	[117]
Alginate with gellan	Bovine articular chondrocytes	Extrusion bioprinting	Tensile modulus: 116–230 kPa	80–96% cell viability. Cartilage ECM deposition Chondrocytes proliferation	[118]
Methacrylated HA with diacrylated Pluronic	Bovine articular chondrocytes	Inkjet bioprinting	Compressive modulus: 1.5–6.5 kPa	62–86% cell viability	[120]
HA with dextran derived	Equine articular chondrocytes	Extrusion bioprinting	Ultimate compressive stress: 100–160 kPa	>75% cell viability	[121]
GelMA with HA	Equine articular chondrocytes	Inkjet bioprinting	Compressive modulus: 5–180 kPa	>73% cell viability	[127]
GelMA with HA-methacrylate	Human bone marrow MSCs	Extrusion bioprinting	Compressive modulus: 48–100 kPa	85–95% cell viability.Chondrogenic differentiation	[126]
GelMA	Equine ACPCs/Chondrocytes/MSCs	Inkjet bioprinting	Compressive modulus: 100–187 kPa	>75% cell viability	[125]
PEGDMA	Human articular chondrocytes	Inkjet bioprinting	Compressive modulus: ~400 kPa	89% cell viability Cartilage ECM deposition	[128]
PEG-GelMA	Human MSCs	Inkjet bioprinting	Compressive modulus: ~1 MPa	~80% cell viability Cartilage ECM deposition Chondrogenic differentiation	[129]
Alginate reinforced with PCL	Embryonic chick chondrocytes	Extrusion bioprinting	Not reported	77–85% cell viability Cartilage ECM deposition	[132]
Alginate reinforced with PCL + TGFβ	Human nososeptal chondrocytes	Extrusion bioprinting	Not reported	85% cell viability Cartilage ECM deposition	[131]
Alginate reinforced with PCL	C20A4 human chondrocyte cell line	Extrusion bioprinting	Compressive modulus: 6 MPa	~70% cell viability	[130]

**Table 3:** Overview of publications on 3D printing of hydrogels for bone tissue engineering.

Material(s)	Cell source(s)	Printing method	Mechanical properties	Cytocompatibility	Reference
MeHA	Human BM MSCs	Inkjet bioprinting	Elastic modulus: ~11 kPa	~65% cell viability Osteogenic differentiation	[141]
Agarose with collagen type I	Human BM MSCs	Inkjet bioprinting	Compressive modulus: 18–90 kPa	>98% cell viability Osteogenic differentiation	[142]
Alginate-gelatin	hASCs	Inkjet bioprinting	Not reported	Osteogenic differentiation Bone matrix formation	[143]
Chitosan-HAp	Human osteoblasts	Extrusion printing	Not reported	Good cell attachment and proliferation	[144]
Chitosan-HAp/Alginate-HAp	MC3T3-E1	Inkjet bioprinting	Elastic modulus: 4.6–15 kPa/3.5–19 kPa	>90% cell viability Cell proliferation Osteogenic differentiation	[145]
MeHA with HAp or GelMA with HAp	hASCs	Extrusion bioprinting	Not reported	Osteogenic differentiation Bone matrix formation	[146]
Alginate-gelatin/alginate-gelatin with nano-HAp	hASCs	Extrusion bioprinting	Not reported	>88% cell viability Osteogenic differentiation	[140]
Alginate-polyvinyl alcohol with HAp	MC3T3-E1 cells	Extrusion bioprinting	Compressive modulus: 2.4–10.3 kPa	77–95% cell viability	[147]
Chitosan reinforced with PCL	Rabbit BM MSCs	Extrusion bioprinting	Compressive strength: 6.7 MPa	Osteogenic differentiation Bone matrix formation	[149]
HA and Gelatin reinforced with PCL/TCP	Human amniotic fluid-SCs	Extrusion bioprinting	Not reported	91% cell viability Osteogenic differentiation Bone matrix formation	[150]
MeHA and GelMA reinforced with PCL/HAp	Stromal vascular fraction derived cells	Extrusion bioprinting	Not reported	Osteogenic differentiation	[151]

## References

[1] Pisani P., Renna M.D., Conversano F., Casciaro E., Di Paola M., Quarta E., Muratore M., Casciaro S. (2016). Major osteoporotic fragility fractures: Risk factor updates and societal impact. World J. Orthop..

[2] Murphy L., Helmick C.G. (2012). The Impact of Osteoarthritis in the United States. Orthop. Nurs..

[3] Tozzi G., De Mori A., Oliveira A., Roldo M. (2016). Composite hydrogels for bone regeneration. Materials (Basel).

[4] Liu M., Zeng X., Ma C., Yi H., Ali Z., Mou X., Li S., Deng Y., He N. (2017). Injectable hydrogels for cartilage and bone tissue engineering. Bone Res..

[5] Kim T.G., Shin H., Lim D.W. (2012). Biomimetic Scaffolds for Tissue Engineering. Adv. Funct. Mater..

[6] Khan W.S., Malik A. (2012). Stem Cell Therapy and Tissue Engineering Applications for Cartilage Regeneration. Curr. Stem Cell Res. Ther..

[7] Grottkau B.E., Lin Y. (2013). Osteogenesis of Adipose-Derived Stem Cells. Bone Res..

[8] Bush J.R., Liang H., Dickinson M., Botchwey E.A. (2016). Xylan hemicellulose improves chitosan hydrogel for bone tissue regeneration. Polym. Adv. Technol..

[9] Trickey W.R., Lee G.M., Guilak F. (2000). Viscoelastic properties of chondrocytes from normal and osteoarthritic human cartilage. J. Orthop. Res..

[10] Pal S. (2014). Design of Artificial Human Joints & Organs.

[11] Mobasheri A., Csaki C., Clutterbuck A.L., Rahmanzadeh M., Shakibaei M. (2009). Mesenchymal stem cells in connective tissue engineering and regenerative medicine: Applications in cartilage repair and osteoarthritis therapy. Histol. Histopathol..

[12] Izadifar Z., Chen X., Kulyk W. (2012). Strategic Design and Fabrication of Engineered Scaffolds for Articular Cartilage Repair. J. Funct. Biomater..

[13] Reddi A.H., Becerra J., Andrades J.A. (2011). Nanomaterials and hydrogel scaffolds for articular cartilage regeneration. Tissue Eng. B.

[14] Hall B.K. (2015). Bones and Cartilage.

[15] Benders K.E.M., van Weeren P.R., Badylak S.F., Saris D.B.F., Dhert W.J.A., Malda J. (2013). Extracellular matrix scaffolds for cartilage and bone regeneration. Trends Biotechnol..

[16] Hadjidakis D.J., Androulakis I.I. (2006). Bone Remodeling. Ann. N. Y. Acad. Sci..

[17] Kini U., Nandeesh B.N., Fogelman I., Gnanasegaran G., van der Wall H. (2012). Physiology of Bone Formation, Remodeling, and Metabolism. Radionuclide and Hybrid Bone Imaging.

[18] Athanasiou K.A., Zhu C., Lanctot D.R., Agrawal C.M., Wang X. (2000). Fundamentals of biomechanics in tissue engineering of bone. Tissue Eng..

[19] Florencio-Silva R., Rodrigues da Silva Sasso G., Sasso-Cerri E., Simoes M.J., Cerri P.S. (2015). Biology of Bone Tissue: Structure, Function, and Factors that Influence Bone Cells. BioMed Res. Int..

[20] Adler C.-P., Adler C.-P. (2000). Bones and Bone Tissue. Bone Diseases: Macroscopic, Histological, and Radiological Diagnosis of Structural Changes in the Skeleton.

[21] Rho J.Y., Ashman R.B., Turner C.H. (2018). Young’s modulus of trabecular and cortical bone material: Ultrasonic and microtensile measurements. J. Biomech..

[22] Wu S., Liu X., Yeung K.W.K., Liu C., Yang X. (2014). Biomimetic porous scaffolds for bone tissue engineering. Mater. Sci. Eng. R Rep..

[23] Fratzl P., Weinkamer R. (2007). Nature’s hierarchical materials. Prog. Mater. Sci..

[24] Kane R., Ma P.X. (2013). Mimicking the nanostructure of bone matrix to regenerate bone. Mater. Today.

[25] Rho J.Y., Kuhn-Spearing L., Zioupos P. (1998). Mechanical properties and the hierarchical structure of bone. Med. Eng. Phys..

[26] Zhang W., Zhu Y., Li J., Guo Q., Peng J., Liu S., Yang J., Wang Y. (2015). Cell-Derived Extracellular Matrix: Basic Characteristics and Current Applications in Orthopedic Tissue Engineering. Tissue Eng. B.

[27] Blair H.C., Sun L., Kohanski R.A. (2007). Balanced Regulation of Proliferation, Growth, Differentiation, and Degradation in Skeletal Cells. Ann. N. Y. Acad. Sci..

[28] Sims N.A., Gooi J.H. (2008). Bone remodeling: Multiple cellular interactions required for coupling of bone formation and resorption. Semin. Cell Dev. Biol..

[29] Oryan A., Monazzah S., Bigham-Sadegh A. (2015). Bone injury and fracture healing biology. Biomed. Environ. Sci..

[30] Schubert T., Lafont S., Beaurin G., Grisay G., Behets C., Gianello P., Dufrane D. (2013). Critical size bone defect reconstruction by an autologous 3D osteogenic-like tissue derived from differentiated adipose MSCs. Biomaterials.

[31] Giannoudis P.V., Einhorn T.A., Marsh D. (2018). Fracture healing: The diamond concept. Injury.

[32] Campana V., Milano G., Pagano E., Barba M., Cicione C., Salonna G., Lattanzi W., Logroscino G. (2014). Bone substitutes in orthopaedic surgery: From basic science to clinical practice. J. Mater. Sci. Mater. Med..

[33] Wang W., Yeung K.W.K. (2017). Bone grafts and biomaterials substitutes for bone defect repair: A review. Bioact. Mater..

[34] Stevens M.M. (2008). Biomaterials for bone tissue engineering. Mater. Today.

[35] Nauth A., Ristevski B., Li R., Schemitsch E.H. (2011). Growth factors and bone regeneration: How much bone can we expect?. Injury.

[36] Agarwal R., Garcia A.J. (2015). Biomaterial strategies for engineering implants for enhanced osseointegration and bone repair. Adv. Drug Deliv. Rev..

[37] Roseti L., Parisi V., Petretta M., Cavallo C., Desando G., Bartolotti I., Grigolo B. (2017). Scaffolds for Bone Tissue Engineering: State of the art and new perspectives. Mater. Sci. Eng. C.

[38] Younger E.M., Chapman M.W. (1989). Morbidity at bone graft donor sites. J. Orthop. Trauma.

[39] García-Gareta E., Coathup M.J., Blunn G.W. (2015). Osteoinduction of bone grafting materials for bone repair and regeneration. Bone.

[40] Yunus Basha R., Sampath Kumar T.S., Doble M. (2015). Design of biocomposite materials for bone tissue regeneration. Mater. Sci. Eng. C.

[41] Dabrowski B., Swieszkowski W., Godlinski D., Kurzydlowski K.J. (2010). Highly porous titanium scaffolds for orthopaedic applications. J. Biomed. Mater. Res. B Appl. Biomater..

[42] Sarkar S.K., Lee B.T. (2015). Hard tissue regeneration using bone substitutes: An update on innovations in materials. Korean J. Intern. Med..

[43] Atala A., Kasper F.K., Mikos A.G. (2012). Engineering complex tissues. Sci. Transl. Med..

[44] Amini A.R., Laurencin C.T., Nukavarapu S.P. (2012). Bone Tissue Engineering: Recent Advances and Challenges. Crit. Rev. Biomed. Eng..

[45] Henkel J., Woodruff M.A., Epari D.R., Steck R., Glatt V., Dickinson I.C., Choong P.F.M., Schuetz M.A., Hutmacher D.W. (2013). Bone Regeneration Based on Tissue Engineering Conceptions—A 21st Century Perspective. Bone Res..

[46] Khorshidi S., Karkhaneh A. (2017). A review on gradient hydrogel/fiber scaffolds for osteochondral regeneration. J. Tissue Eng. Regen. Med..

[47] Nukavarapu S.P., Dorcemus D.L. (2013). Osteochondral tissue engineering: Current strategies and challenges. Biotechnol. Adv..

[48] Nooeaid P., Salih V., Beier J.P., Boccaccini A.R. (2012). Osteochondral tissue engineering: Scaffolds, stem cells and applications. J. Cell. Mol. Med..

[49] Gomoll A.H., Madry H., Knutsen G., van Dijk N., Seil R., Brittberg M., Kon E. (2010). The subchondral bone in articular cartilage repair: Current problems in the surgical management. Knee Surg. Sports Traumatol. Arthrosc..

[50] Seliktar D. (2012). Designing cell-compatible hydrogels for biomedical applications. Science.

[51] Zhang L., Xia K., Lu Z., Li G., Chen J., Deng Y., Li S., Zhou F., He N. (2014). Efficient and Facile Synthesis of Gold Nanorods with Finely Tunable Plasmonic Peaks from Visible to Near-IR Range. Chem. Mater..

[52] Peppas N.A., Bures P., Leobandung W., Ichikawa H. (2000). Hydrogels in pharmaceutical formulations. Eur. J. Pharm. Biopharm..

[53] Vega S., Kwon M., Burdick J. (2017). Recent advances in hydrogels for cartilage tissue engineering. Eur. Cells Mater..

[54] Visser J., Melchels F.P.W., Jeon J.E., van Bussel E.M., Kimpton L.S., Byrne H.M., Dhert W.J.A., Dalton P.D., Hutmacher D.W., Malda J. (2015). Reinforcement of hydrogels using three-dimensionally printed microfibres. Nat. Commun..

[55] Vedadghavami A., Minooei F., Mohammadi M.H., Khetani S., Rezaei Kolahchi A., Mashayekhan S., Sanati-Nezhad A. (2017). Manufacturing of hydrogel biomaterials with controlled mechanical properties for tissue engineering applications. Acta Biomater..

[56] Snyder T.N., Madhavan K., Intrator M., Dregalla R.C., Park D. (2014). A fibrin/hyaluronic acid hydrogel for the delivery of mesenchymal stem cells and potential for articular cartilage repair. J. Biol. Eng..

[57] Guo Y., Yuan T., Xiao Z., Tang P., Xiao Y., Fan Y., Zhang X. (2012). Hydrogels of collagen/chondroitin sulfate/hyaluronan interpenetrating polymer network for cartilage tissue engineering. J. Mater. Sci. Mater. Med..

[58] Yanagawa F., Sugiura S., Kanamori T. (2016). Hydrogel microfabrication technology toward three dimensional tissue engineering. Regen. Ther..

[59] Da Costa F.F.P., Araujo E.S., Nascimineto M.L.F., de Oliveira H.P. (2015). Electrospun Fibers of Enteric Polymer for Controlled Drug Delivery. Int. J. Polym. Sci..

[60] Scholten E., Bromberg L., Rutledge G.C., Hatton T.A. (2011). Electrospun Polyurethane Fibers for Absorption of Volatile Organic Compounds from Air. ACS Appl. Mater. Interfaces.

[61] Li G., Zhang T., Li M., Fu N., Fu Y., Ba K., Deng S., Jiang Y., Hu J., Peng Q. (2014). Electrospun fibers for dental and craniofacial applications. Curr. Stem Cell Res. Ther..

[62] Hu X., Liu S., Zhou G., Huang Y., Xie Z., Jing X. (2014). Electrospinning of polymeric nanofibers for drug delivery applications. J. Control. Release.

[63] Shi X., Zhou W., Ma D., Ma Q., Bridges D., Ma Y., Hu A. (2015). Electrospinning of Nanofibers and Their Applications for Energy Devices. J. Nanomater..

[64] Zhu P., Lin A., Tang X., Lu X., Zheng J., Zheng G., Lei T. (2016). Fabrication of three-dimensional nanofibrous macrostructures by electrospinning. AIP Adv..

[65] Haider A., Haider S., Kang I.-K. (2015). A comprehensive review summarizing the effect of electrospinning parameters and potential applications of nanofibers in biomedical and biotechnology. Arab. J. Chem..

[66] Ray S.S., Chen S.-S., Li C.-W., Nguyen N.C., Nguyen H.T. (2016). A comprehensive review: Electrospinning technique for fabrication and surface modification of membranes for water treatment application. RSC Adv..

[67] Erdem-Kuruca S., Kayaman-Apohan N., Oktay B. (2014). Fabrication of nanofiber mats from electrospinning of functionalized polymers. IOP Conf. Ser. Mater. Sci. Eng..

[68] Thompson C.J., Chase G.G., Yarin A.L., Reneker D.H. (2007). Effects of parameters on nanofiber diameter determined from electrospinning model. Polymer (Guildford).

[69] Sadat-Shojai M., Khorasani M.-T., Jamshidi A. (2016). A new strategy for fabrication of bone scaffolds using electrospun nano-HAp/PHB fibers and protein hydrogels. Chem. Eng. J..

[70] Bosworth L.A., Turner L.-A., Cartmell S.H. (2013). State of the art composites comprising electrospun fibres coupled with hydrogels: A review. Nanomedicine.

[71] Coburn J., Gibson M., Bandalini P.A., Laird C., Mao H.-Q., Moroni L., Seliktar D., Elisseeff J. (2011). Biomimetics of the Extracellular Matrix: An Integrated Three-Dimensional Fiber-Hydrogel Composite for Cartilage Tissue Engineering. Smart Struct. Syst..

[72] Mirahmadi F., Tafazzoli-Shadpour M., Shokrgozar M.A., Bonakdar S. (2013). Enhanced mechanical properties of thermosensitive chitosan hydrogel by silk fibers for cartilage tissue engineering. Mater. Sci. Eng. C.

[73] Mohabatpour F., Karkhaneh A., Sharifi A.M. (2016). A hydrogel/fiber composite scaffold for chondrocyte encapsulation in cartilage tissue regeneration. RSC Adv..

[74] Coburn J.M., Gibson M., Monagle S., Patterson Z., Elisseeff J.H. (2012). Bioinspired nanofibers support chondrogenesis for articular cartilage repair. Proc. Natl. Acad. Sci. USA.

[75] Bas O., De-Juan-Pardo E.M., Meinert C., D’Angella D., Baldwin J.G., Bray L.J., Wellard R.M., Kollmannsberger S., Rank E., Werner C. (2017). Biofabricated soft network composites for cartilage tissue engineering. Biofabrication.

[76] Tonsomboon K., Butcher A.L., Oyen M.L. (2017). Strong and tough nanofibrous hydrogel composites based on biomimetic principles. Mater. Sci. Eng. C.

[77] He Q., Wang Z., Yan Y., Zheng J., Cai S. (2016). Polymer nanofiber reinforced double network gel composite: Strong, tough and transparent. Extrem. Mech. Lett..

[78] Wright L.D., McKeon-Fischer K.D., Cui Z., Nair L.S., Freeman J.W. (2014). PDLA/PLLA and PDLA/PCL nanofibers with a chitosan-based hydrogel in composite scaffolds for tissue engineered cartilage. J. Tissue Eng. Regen. Med..

[79] Bas O., De-Juan-Pardo E.M., Chhaya M.P., Wunner F.M., Jeon J.E., Klein T.J., Hutmacher D.W. (2015). Enhancing structural integrity of hydrogels by using highly organised melt electrospun fibre constructs. Eur. Polym. J..

[80] Formica F.A., Öztürk E., Hess S.C., Stark W.J., Maniura-Weber K., Rottmar M., Zenobi-Wong M. (2016). A Bioinspired Ultraporous Nanofiber-Hydrogel Mimic of the Cartilage Extracellular Matrix. Adv. Healthc. Mater..

[81] Liu W., Zhan J., Su Y., Wu T., Ramakrishna S., Liao S., Mo X. (2014). Injectable hydrogel incorporating with nanoyarn for bone regeneration. J. Biomater. Sci. Polym. Ed..

[82] Teo W.-E., Gopal R., Ramaseshan R., Fujihara K., Ramakrishna S. (2007). A dynamic liquid support system for continuous electrospun yarn fabrication. Polymer (Guildford).

[83] Kolambkar Y.M., Dupont K.M., Boerckel J.D., Huebsch N., Mooney D.J., Hutmacher D.W., Guldberg R.E. (2011). An alginate-based hybrid system for growth factor delivery in the functional repair of large bone defects. Biomaterials.

[84] Filova E., Rampichova M., Litvinec A., Drzik M., Mickova A., Buzgo M., Kostakova E., Martinova L., Usvald D., Prosecka E. (2013). A cell-free nanofiber composite scaffold regenerated osteochondral defects in miniature pigs. Int. J. Pharm..

[85] Chua C.K., Leong K.F., Lim C.S. (2010). Rapid Prototyping. Principles and Applications.

[86] Peltola S.M., Melchels F.P.W., Grijpma D.W., Kellomaki M. (2008). A review of rapid prototyping techniques for tissue engineering purposes. Ann. Med..

[87] Sachlos E., Czernuszka J.T. (2003). Making tissue engineering scaffolds work. Review: The application of solid freeform fabrication technology to the production of tissue engineering scaffolds. Eur. Cell Mater..

[88] Do A.-V., Khorsand B., Geary S.M., Salem A.K. (2015). 3D Printing of Scaffolds for Tissue Regeneration Applications. Adv. Healthc. Mater..

[89] Billiet T., Vandenhaute M., Schelfhout J., Van Vlierberghe S., Dubruel P. (2012). A review of trends and limitations in hydrogel-rapid prototyping for tissue engineering. Biomaterials.

[90] Melchels F.P.W., Feijen J., Grijpma D.W. (2010). A review on stereolithography and its applications in biomedical engineering. Biomaterials.

[91] Liska R., Schuster M., Inführ R., Turecek C., Fritscher C., Seidl B., Schmidt V., Kuna L., Haase A., Varga F. (2007). Photopolymers for rapid prototyping. J. Coat. Technol. Res..

[92] Dhariwala B., Hunt E., Boland T. (2004). Rapid prototyping of tissue-engineering constructs, using photopolymerizable hydrogels and stereolithography. Tissue Eng..

[93] Khalil S., Nam J., Sun W. (2005). Multi-nozzle deposition for construction of 3D biopolymer tissue scaffolds. Rapid Prototyp. J..

[94] Liu L., Xiong Z., Yan Y., Zhang R., Wang X., Jin L. (2009). Multinozzle low-temperature deposition system for construction of gradient tissue engineering scaffolds. J. Biomed. Mater. Res. B Appl. Biomater..

[95] Vozzi G., Ahluwalia A. (2007). Microfabrication for tissue engineering: Rethinking the cells-on-a scaffold approach. J. Mater. Chem..

[96] Sachs E., Cima M., Cornie J. (1990). Three-Dimensional Printing: Rapid Tooling and Prototypes Directly from a CAD Model. CIRP Ann..

[97] Nakamura M., Kobayashi A., Takagi F., Watanabe A., Hiruma Y., Ohuchi K., Iwasaki Y., Horie M., Morita I., Takatani S. (2005). Biocompatible inkjet printing technique for designed seeding of individual living cells. Tissue Eng..

[98] Chia H.N., Wu B.M. (2015). Recent advances in 3D printing of biomaterials. J. Biol. Eng..

[99] Mandrycky C., Wang Z., Kim K., Kim D. (2016). 3D bioprinting for engineering complex tissues. Biotechnol. Adv..

[100] Pereira R.F., Bartolo P.J. (2015). 3D bioprinting of photocrosslinkable hydrogel constructs. J. Appl. Polym. Sci..

[101] Guvendiren M., Lu H.D., Burdick J.A. (2012). Shear-thinning hydrogels for biomedical applications. Soft Matter.

[102] Stanton M.M., Samitier J., Sanchez S. (2015). Bioprinting of 3D hydrogels. Lab Chip.

[103] Holzl K., Lin S., Tytgat L., Van Vlierberghe S., Gu L., Ovsianikov A. (2016). Bioink properties before, during and after 3D bioprinting. Biofabrication.

[104] Zhang X., Zhang Y. (2015). Tissue Engineering Applications of Three-Dimensional Bioprinting. Cell Biochem. Biophys..

[105] Malda J., Visser J., Melchels F.P., Jüngst T., Hennink W.E., Dhert W.J.A., Groll J., Hutmacher D.W. (2013). 25th Anniversary Article: Engineering Hydrogels for Biofabrication. Adv. Mater..

[106] Khalil S., Sun W. (2009). Bioprinting endothelial cells with alginate for 3D tissue constructs. J. Biomech. Eng..

[107] Tirella A., Orsini A., Vozzi G., Ahluwalia A. (2009). A phase diagram for microfabrication of geometrically controlled hydrogel scaffolds. Biofabrication.

[108] Luo Y., Zhai D., Huan Z., Zhu H., Xia L., Chang J., Wu C. (2015). Three-Dimensional Printing of Hollow-Struts-Packed Bioceramic Scaffolds for Bone Regeneration. ACS Appl. Mater. Interfaces.

[109] Osterbur L.W. (2013). 3D Printing of Hyaluronic Acid Scaffolds for Tissue Engineering Applications.

[110] Zhao S., Zhang J., Zhu M., Zhang Y., Liu Z., Tao C., Zhu Y., Zhang C. (2015). Three-dimensional printed strontium-containing mesoporous bioactive glass scaffolds for repairing rat critical-sized calvarial defects. Acta Biomater..

[111] Jakus A.E., Taylor S.L., Geisendorfer N.R., Dunand D.C., Shah R.N. (2015). Metallic Architectures from 3D-Printed Powder-Based Liquid Inks. Adv. Funct. Mater..

[112] You F., Eames B.F., Chen X. (2017). Application of Extrusion-Based Hydrogel Bioprinting for Cartilage Tissue Engineering. Int. J. Mol. Sci..

[113] You F., Wu X., Zhu N., Lei M., Eames B.F., Chen X. (2016). 3D Printing of Porous Cell-Laden Hydrogel Constructs for Potential Applications in Cartilage Tissue Engineering. ACS Biomater. Sci. Eng..

[114] Klein T.J., Rizzi S.C., Reichert J.C., Georgi N., Malda J., Schuurman W., Crawford R.W., Hutmacher D.W. (2009). Strategies for Zonal Cartilage Repair using Hydrogels. Macromol. Biosci..

[115] Markstedt K., Mantas A., Tournier I., Martínez Ávila H., Hägg D., Gatenholm P. (2015). 3D Bioprinting Human Chondrocytes with Nanocellulose–Alginate Bioink for Cartilage Tissue Engineering Applications. Biomacromolecules.

[116] Möller T., Amoroso M., Hägg D., Brantsing C., Rotter N., Apelgren P., Lindahl A., Kölby L., Gatenholm P. (2017). In Vivo Chondrogenesis in 3D Bioprinted Human Cell-laden Hydrogel Constructs. Plast. Reconstr. Surg. Glob. Open.

[117] Apelgren P., Amoroso M., Lindahl A., Brantsing C., Rotter N., Gatenholm P., Kölby L. (2017). Chondrocytes and stem cells in 3D-bioprinted structures create human cartilage in vivo. PLoS ONE.

[118] Kesti M., Eberhardt C., Pagliccia G., Kenkel D., Grande D., Boss A., Zenobi-Wong M. (2015). Bioprinting Complex Cartilaginous Structures with Clinically Compliant Biomaterials. Adv. Funct. Mater..

[119] Burdick J.A., Prestwich G.D. (2011). Hyaluronic Acid Hydrogels for Biomedical Applications. Adv. Mater..

[120] Müller M., Becher J., Schnabelrauch M., Zenobi-Wong M. (2015). Nanostructured Pluronic hydrogels as bioinks for 3D bioprinting. Biofabrication.

[121] Pescosolido L., Schuurman W., Malda J., Matricardi P., Alhaique F., Coviello T., van Weeren P.R., Dhert W.J.A., Hennink W.E., Vermonden T. (2011). Hyaluronic Acid and Dextran-Based Semi-IPN Hydrogels as Biomaterials for Bioprinting. Biomacromolecules.

[122] Wang X., Tuomi J., Mäkitie A.A., Paloheimo K.-S., Partanen J., Yliperttula M., Pignatello R. (2013). The Integrations of Biomaterials and Rapid Prototyping Techniques for Intelligent Manufacturing of Complex Organs.

[123] Ifkovits J.L., Burdick J.A. (2007). Review: Photopolymerizable and degradable biomaterials for tissue engineering applications. Tissue Eng..

[124] Billiet T., Gevaert E., De Schryver T., Cornelissen M., Dubruel P. (2014). The 3D printing of gelatin methacrylamide cell-laden tissue-engineered constructs with high cell viability. Biomaterials.

[125] Levato R., Webb W.R., Otto I.A., Mensinga A., Zhang Y., van Rijen M., van Weeren R., Khan I.M., Malda J. (2017). The bio in the ink: Cartilage regeneration with bioprintable hydrogels and articular cartilage-derived progenitor cells. Acta Biomater..

[126] Costantini M., Idaszek J., Szöke K., Jaroszewicz J., Dentini M., Barbetta A., Brinchmann J.E., Święszkowski W. (2016). 3D bioprinting of BM-MSCs-loaded ECM biomimetic hydrogels for in vitro neocartilage formation. Biofabrication.

[127] Schuurman W., Levett P.A., Pot M.W., van Weeren P.R., Dhert W.J.A., Hutmacher D.W., Melchels F.P.W., Klein T.J., Malda J. (2013). Gelatin-Methacrylamide Hydrogels as Potential Biomaterials for Fabrication of Tissue-Engineered Cartilage Constructs. Macromol. Biosci..

[128] Cui X., Breitenkamp K., Finn M.G., Lotz M., D’Lima D.D. (2012). Direct Human Cartilage Repair Using Three-Dimensional Bioprinting Technology. Tissue Eng. Part A.

[129] Gao G., Schilling A.F., Hubbell K., Yonezawa T., Truong D., Hong Y., Dai G., Cui X. (2015). Improved properties of bone and cartilage tissue from 3D inkjet-bioprinted human mesenchymal stem cells by simultaneous deposition and photocrosslinking in PEG-GelMA. Biotechnol. Lett..

[130] Schuurman W., Khristov V., Pot M.W., van Weeren P.R., Dhert W.J., Malda J. (2011). Bioprinting of hybrid tissue constructs with tailorable mechanical properties. Biofabrication.

[131] Kundu J., Shim J.-H., Jang J., Kim S.-W., Cho D.-W. (2015). An additive manufacturing-based PCL-alginate-chondrocyte bioprinted scaffold for cartilage tissue engineering. J. Tissue Eng. Regen. Med..

[132] Izadifar Z., Chang T., Kulyk W., Chen X., Eames B.F. (2016). Analyzing Biological Performance of 3D-Printed, Cell-Impregnated Hybrid Constructs for Cartilage Tissue Engineering. Tissue Eng. Part C Methods.

[133] Murphy S.V., Skardal A., Atala A. (2013). Evaluation of hydrogels for bio-printing applications. J. Biomed. Mater. Res. A.

[134] Zhu J., Marchant R.E. (2011). Design properties of hydrogel tissue-engineering scaffolds. Expert Rev. Med. Devices.

[135] Maas M., Hess U., Rezwan K. (2014). The contribution of rheology for designing hydroxyapatite biomaterials. Curr. Opin. Colloid Interface Sci..

[136] Hayrapetyan A., Bongio M., Leeuwenburgh S.C.G., Jansen J.A., van den Beucken J.J.J.P. (2016). Effect of Nano-HA/Collagen Composite Hydrogels on Osteogenic Behavior of Mesenchymal Stromal Cells. Stem Cell Rev. Rep..

[137] Wust S., Godla M.E., Muller R., Hofmann S. (2014). Tunable hydrogel composite with two-step processing in combination with innovative hardware upgrade for cell-based three-dimensional bioprinting. Acta Biomater..

[138] Sadat-Shojai M., Khorasani M.-T., Jamshidi A. (2015). 3-Dimensional cell-laden nano-hydroxyapatite/protein hydrogels for bone regeneration applications. Mater. Sci. Eng. C Mater. Biol. Appl..

[139] Thakur T., Xavier J.R., Cross L., Jaiswal M.K., Mondragon E., Kaunas R., Gaharwar A.K. (2016). Photocrosslinkable and elastomeric hydrogels for bone regeneration. J. Biomed. Mater. Res. A.

[140] Wang X.-F., Lu P.-J., Song Y., Sun Y.-C., Wang Y.-G., Wang Y. (2016). Nano hydroxyapatite particles promote osteogenesis in a three-dimensional bio-printing construct consisting of alginate/gelatin/hASCs. RSC Adv..

[141] Poldervaart M.T., Goversen B., de Ruijter M., Abbadessa A., Melchels F.P.W., Öner F.C., Dhert W.J.A., Vermonden T., Alblas J. (2017). 3D bioprinting of methacrylated hyaluronic acid (MeHA) hydrogel with intrinsic osteogenicity. PLoS ONE.

[142] Duarte Campos D.F., Blaeser A., Buellesbach K., Sen K.S., Xun W., Tillmann W., Fischer H. (2016). Bioprinting Organotypic Hydrogels with Improved Mesenchymal Stem Cell Remodeling and Mineralization Properties for Bone Tissue Engineering. Adv. Healthc. Mater..

[143] Wang X.-F., Song Y., Liu Y.-S., Sun Y., Wang Y., Wang Y., Lyu P.-J. (2016). Osteogenic Differentiation of Three-Dimensional Bioprinted Constructs Consisting of Human Adipose-Derived Stem Cells In Vitro and In Vivo. PLoS ONE.

[144] Ang T.H., Sultana F.S.A., Hutmacher D.W., Wong Y.S., Fuh J.Y.H., Mo X.M., Loh H.T., Burdet E., Teoh S.H. (2002). Fabrication of 3D chitosan-hydroxyapatite scaffolds using a robotic dispensing system. Mater. Sci. Eng. C.

[145] Demirtaş T.T., Irmak G., Gümüşderelioğlu M. (2017). A bioprintable form of chitosan hydrogel for bone tissue engineering. Biofabrication.

[146] Wenz A., Borchers K., Tovar G.E.M., Kluger P.J. (2017). Bone matrix production in hydroxyapatite-modified hydrogels suitable for bone bioprinting. Biofabrication.

[147] Bendtsen S.T., Quinnell S.P., Wei M. (2017). Development of a novel alginate-polyvinyl alcohol-hydroxyapatite hydrogel for 3D bioprinting bone tissue engineered scaffolds. J. Biomed. Mater. Res. Part A.

[148] Ciardelli G., Chiono V., Vozzi G., Pracella M., Ahluwalia A., Barbani N., Cristallini C., Giusti P. (2005). Blends of poly-(epsilon-caprolactone) and polysaccharides in tissue engineering applications. Biomacromolecules.

[149] Dong L., Wang S.-J., Zhao X.-R., Zhu Y.-F., Yu J.-K. (2017). 3D-Printed Poly(ε-caprolactone) Scaffold Integrated with Cell-laden Chitosan Hydrogels for Bone Tissue Engineering. Sci. Rep..

[150] Kang H.-W., Lee S.J., Ko I.K., Kengla C., Yoo J.J., Atala A. (2016). A 3D bioprinting system to produce human-scale tissue constructs with structural integrity. Nat. Biotechnol..

[151] Kuss M.A., Harms R., Wu S., Wang Y., Untrauer J.B., Carlson M.A., Duan B. (2017). Short-term hypoxic preconditioning promotes prevascularization in 3D bioprinted bone constructs with stromal vascular fraction derived cells. Rsc Adv..

[152] Bracaglia L.G., Messina M.J., Winston S., Kuo C.-Y., Lerman M., Fisher J.P. (2017). 3D Printed Pericardium Hydrogels to Promote Wound Healing in Vascular Applications. Biomacromolecules.

[153] Fedorovich N.E., Schuurman W., Wijnberg H.M., Prins H.-J., van Weeren P.R., Malda J., Alblas J., Dhert W.J.A. (2012). Biofabrication of Osteochondral Tissue Equivalents by Printing Topologically Defined, Cell-Laden Hydrogel Scaffolds. Tissue Eng. C.

[154] Levato R., Visser J., Planell J.A., Engel E., Malda J., Mateos-Timoneda M.A. (2014). Biofabrication of tissue constructs by 3D bioprinting of cell-laden microcarriers. Biofabrication.

[155] Castro N.J., O’Brien J., Zhang L.G. (2015). Integrating Biologically Inspired Nanomaterials and Table-top Stereolithography for 3D Printed Biomimetic Osteochondral Scaffolds. Nanoscale.

[156] Gillette B.M., Jensen J.A., Tang B., Yang G.J., Bazargan-Lari A., Zhong M., Sia S.K. (2008). In situ collagen assembly for integrating microfabricated three-dimensional cell-seeded matrices. Nat. Mater..

[157] Sahoo S., Chung C., Khetan S., Burdick J.A. (2008). Hydrolytically Degradable Hyaluronic Acid Hydrogels with Controlled Temporal Structures. Biomacromolecules.

[158] Park J.Y., Choi J.-C., Shim J.-H., Lee J.-S., Park H., Kim S.W., Doh J., Cho D.-W. (2014). A comparative study on collagen type I and hyaluronic acid dependent cell behavior for osteochondral tissue bioprinting. Biofabrication.

[159] Shim J.-H., Jang K.-M., Hahn S.K., Park J.Y., Jung H., Oh K., Park K.M., Yeom J., Park S.H., Kim S.W. (2016). Three-dimensional bioprinting of multilayered constructs containing human mesenchymal stromal cells for osteochondral tissue regeneration in the rabbit knee joint. Biofabrication.

[160] Lee S.J., Heo D.N., Park J.S., Kwon S.K., Lee J.H., Lee J.H., Kim W.D., Kwon I.K., Park S.A. (2015). Characterization and preparation of bio-tubular scaffolds for fabricating artificial vascular grafts by combining electrospinning and a 3D printing system. Phys. Chem. Chem. Phys..

[161] Yu Y., Hua S., Yang M., Fu Z., Teng S., Niu K., Zhao Q., Yi C. (2016). Fabrication and characterization of electrospinning/3D printing bone tissue engineering scaffold. RSC Adv..

[162] Naghieh S., Foroozmehr E., Badrossamay M., Kharaziha M. (2017). Combinational processing of 3D printing and electrospinning of hierarchical poly(lactic acid)/gelatin-forsterite scaffolds as a biocomposite: Mechanical and biological assessment. Mater. Des..

